# Resistance of Particleboards Made from Fast-Growing Wood Species to Subterranean Termite Attack

**DOI:** 10.3390/insects3020532

**Published:** 2012-05-29

**Authors:** Dede Hermawan, Yusuf S. Hadi, Esi. Fajriani, Muhamad Y. Massijaya, Nurwati Hadjib

**Affiliations:** 1 Bogor Agricultural University, Bogor 16680, Indonesia; E-Mails: dedemjmr@yahoo.co.id (D.H.); fajriani_esi@yahoo.co.id (E.F.); mymassijaya@yahoo.co.id (M.Y.M.); 2 Forest Products Research Institute, Bogor 16680, Indonesia; E-Mail: nurwati_hadjib@yahoo.com

**Keywords:** particleboard, fast growing species, subterranean termite, mass loss, feeding rate

## Abstract

Laboratory-made particleboards were tested for their resistance to subterranean termite, *Coptotermes curvignathus *Holmgren (Order Isoptera, Family Termitidae) by Indonesian standard SNI 01.7207–2006, during four weeks and at the end of the test their mass loss percentage and feeding rate were determined. Particleboards consisted of: jabon (*Anthocephalus cadamba*, Family Rubiacea) with a density of 0.41 g/cm^3^; sungkai (*Peronema canescens*, Family Verbenaceae) with a density of 0.46 g/cm^3^; mangium (*Acacia mangium*, Family Rhamnaceae) with a density of 0.60 g/cm^3^ separately and the three species mixture at a rate of 1:1:1. Densities of the boards were targetted at 0.60 g/cm^3^ and 0.80 g/cm^3^ by using 12% urea formaldehyde as binder with 2% paraffin as additive based on oven dry wood particle weight. The hand-formed mats and hot-pressing at 130 °C and 2.45 MPa for 10 min were applied. The results showed that particleboards density did not affect mass loss and feeding rate, but the particleboards made from higher density wood resulted in higher resistance to subterranean termite attack. The most resistant particleboards were made of magium, followed by sungkai, mixed species, and jabon.

## 1. Introduction

Natural forests used to account for 90% of log supply in Indonesia; however there was a drastic change in 2000 when 60% logs were produced from plantation forests [1]. Fast-growing tree species such as mangium (*Acacia mangium*, Family Rhamnaceae), mahogany (*Swietenia macrophylla*, Family Meliacea), pine (*Pinus merkusii*, Family Pinaceae), sengon (*Paraserianthes falcataria*, Family Mimosaseae)*, *sonokeling (*Dalbergia latifolia*, Family Papilionaceae) and sungkai (*Peronema canescens*, Family Verbenaceae) have been planted over approximately 4 million hectares, and these species are harvested every 10–15 years [2]. Recently jabon (*Anthocephalus cadamba*, Family Rubiaceae) has been gaining popularity because of a few favorable characteristics (fast-growing, straight and cylindric stem, fewer knots, light color and density of wood 0.43 g/cm^3^) [3].

Wood from plantation forests generally contains a lot of juvenile wood which is inferior to mature wood in physical-mechanical properties and durability. However, Indonesian houses built from mature wood are not immune to termite attacks. In 1995 the economic losses caused by termites in various buildings amounted to approxiately US$ 200 million [4], and rose up to US$ 200–300 million in 2000 [5]. There is no doubt that there will be increased economic loss in the future, when wood products from plantation forests will be widely used as replacements for those from natural forests.

On the other hand, Massijaya *et al*. mentioned that small diameter logs from fast-growing species offer good prospects for biocomposite products, such as plywood, particleboard, fiberboard, cementboard, glulam, laminated veneer lumber, and these products are expected to meet national and international requirements for physical and mechanical performance [6]. Unfortunately, the durability of biocomposite products is not yet fully known, although it is thought to be similar to that of raw materials.

The purpose of this research was to determine the termite-resistance of particleboards made from fast-growing wood species, especially jabon, sungkai, mangium, and the mixture of the three species by using the Indonesian standardized laboratory test.

## 2. Materials and Methods

### 2.1. Preparation of Particleboards

Particles were prepared from small or less than 30 cm diameter logs of: jabon (*Anthocephalus cadamba*) with a density of 0.41 g/cm^3^; sungkai (*Peronema canescens*) with a density of 0.46 g/cm^3^; and mangium (*Acacia mangium*) with a density of 0.60 g/cm^3^. Particleboards were made from each wood species and the three species mixture at a rate of 1:1:1 by using 12% of urea formaldehyde as a binder and 2% paraffin based on oven dried wood particles weight for improving water resistance. Density targets of the boards were 0.60 g/cm^3^ or medium density particleboard and 0.80 g/cm^3^ or high density particleboard The hand-formed particle mats were hot-pressed at 130 °C temperature and 2.45 Mpa pressure for 10 min to produce boards 30 cm × 30 cm × 1 cm (length × width × thickness respectively).

### 2.2. Subterranean Termite Test

Table 1 summarizes the test method in SNI 01.7207–2006 [7,8]. A board sample was placed in a jam pot, leaning against a side wall with 200 g sand (7% moisture content under water holding capacity of the sand) and 200 healthy and active workers of subterranean termites, *Coptotermes curvignathus *Holmgren (Order Isoptera, Family Termitidae).

**Table 1:** Subterranean termite test method (SNI 01.7207–2006) [8].

Item	Description
General	No-choice test
Test termite species	*Coptotermes curvignathus *Holmgren
Number of termites used per test unit	200 workers
Wood specimens	Species: Not specified for control wood Size: 2.5 cm (L) × 2.5 cm (T) × 0.5 cm (R) (dressed sawn)
Pre- and post-conditioning of wood specimens	Oven-drying at 102 ± 3 °C until no weight change detected
Weathering procedure	Not specified
Test container	Round jam pot (450–500 mL capacity) with a wide-mouth and a bottom area of 25–30 cm^2^
Test assembly	An individual wood specimen buried in 200 g sand at 7% MC below water holding capacity of the sand (or moist sand) in the jam pot so that one of the largest areas of the sample (the widest part) allowed to contact inside vertical wall of the pot

The jam pots were placed in a dark room for 4 weeks, and the bottles were weighted weekly to regulate the moisture content of the sand. At the end of the test, mass loss percent of each particleboard was determined according to SNI 01.7207–2006 [7], and feeding rate was also calculated. Wood samples of each species were included in the test as reference controls.

### 2.3. Data Analysis

Factorial 2 by 4 in completely randomized design was used to analyze the data. The first factor was board density, namely medium density particleboard (0.60 g/cm^3^) and high density particleboard (0.80 g/cm^3^), and the other factor was wood species for particleboard manufacturing, *i.e.*, jabon, sungkai, mangium, and mixture of the three species. The replication of specimen was three, and Duncan’s test was used for further analysis if the factor was significantly different.

## 3. Results and Discussion

### 3.1. Mass Loss

Mass loss percentages and feeding rates of particleboards and wood samples are shown in Table 2. The results of analysis of variance for mass losses and feeding rates are shown in Table 3. With regard to the analysis of variance shown in Table 3, wood species as raw material was classified as highly significantly affected at 1% significant level to particleboard mass loss percentage, and it could be mentioned that higher-density woods tended to be more resistant to *Coptotermes curvignathus *as shown in Table 2. This was similar to the results obtained by Arango *et al*. who exposed six hardwood species to *Reticulitermes flavipes* [9]. Such a tendency was also common to particleboards from higher-density woods, they were more resistant to termite attacks than those from lower-density woods.

**Table 2:** Mass loss and feeding rate of each wood species particleboard and its type.

Respond	Board Type	Jabon	Sungkai	Mangium	Mixed
Mass loss (%)	Medium-Density Particleboard	6.9 ± 1.2	4.5 ± 0.4	4.1 ± 0.3	6.2 ± 1.2
	High-Density Particleboard	5.0 ± 0.4	4.3 ± 0.3	3.6 ± 0.4	5.4 ± 2.5
	Solid Wood	8.4 ± 0.5	3.5 ± 0.6	2.4 ± 0.4	
Feeding rate (µg/termite/day)	Medium Density Particleboard	83.8 ± 11.3	60.6 ± 9.8	53.5 ± 3.4	90.2 ± 31.3
	High Density Particleboard	92.3 ± 7.0	73.6 ± 8.2	67.0 ± 8.5	100.7 ± 50.4
	Solid Wood	89.5 ± 10.4	34.5 ± 6.5	29.3 ± 6.6	

**Table 3:** Analysis of variance for mass loss and feeding rate.

Factor	Mass Loss		Feeding Rate	
	F value	Sign. level	F value	Sign. level
Board density	3.50	0.080	1.57	0.228
Wood species	5.62	0.008 **	3.41	0.043 *
Interaction	0.65	0.593	0.02	0.997

Remarks: * Significantly different at *p* < 5%; ** Highly significantly different at *p* < 1%.

The analysis of variance also showed that particleboard density and interaction of both factors did not affect the mass loss of particleboard (Table 3).

Particleboards from higher-density wood sustained lower mass loss percentage and the board from mangium (wood density 0.60 g/cm^3^) was the most resistant, followed by sungkai (wood density 0.46 g/cm^3^), mixed species, and jabon (wood density 0.41 g/cm^3^). Regarding Duncan’s test, it can be mentioned that jabon wood was the most resistant and differed from sungkai and mangium woods, and both sungkai and mangium woods were not different from each other. Since none of the fast-growing wood species tested and particleboards prepared from them was resistant to termite attacks, it may be needed to treat these wood products chemically, physically or chemically-physically to ensure a longer service life as building components.

### 3.2. Feeding Rate

Feeding rates (µg/termite/day) determined for each wood species as raw material and density of particleboard are shown in Table 2. Statistical analysis also demonstrated the mass losses and feeding rates of particleboards (Table 3), resulting in lower feeding rates being found in particleboards from higher density woods.

In this research, feeding rate was not linear with mass loss percentage because mass loss percentage was calculated based on the amount of board mass removed by termites divided by the initial weight of the board at the same volume of the specimen. Therefore, when density of the board increased, the mass loss percentage decreased, as indicated in Table 2. Since the calculation of feeding rate was based on mass loss divided by total live termite and total tested days, high-density particleboards from jabon, sungkai, and mangium had higher feeding rate values than medium-density particleboards for the same wood species.

## 4. Conclusions

Statistical analysis suggested that the choice of wood species as raw material was relevant in resultant mass losses and feeding rates. Laboratory evaluations further indicated that higher-density wood as raw material for particleboard resulted in higher resistance to subterranean termites, *Coptotermes curvignathus*. Since none of the fast-growing wood species tested and particleboards prepared from them were resistant to termite attacks, it is recommended that these wood products be treated chemically, physically or chemically-physically to ensure a longer service life as building components.
